# A Wrinkling and Etching-Assisted Regrowth Strategy for Large-Area Bilayer Graphene Preparation on Cu

**DOI:** 10.3390/nano13142059

**Published:** 2023-07-12

**Authors:** Qiongyu Li, Tongzhi Liu, You Li, Fang Li, Yanshuai Zhao, Shihao Huang

**Affiliations:** 1 School of Electronic, Electrical Engineering and Physics, Fujian University of Technology, Fuzhou 350118, China; 2 MIIT Key Laboratory of Semiconductor Microstructure and Quantum Sensing, Department of Applied Physics, Nanjing University of Science and Technology, Nanjing 210094, China

**Keywords:** graphene, growth mechanism, large area, wrinkle

## Abstract

Bilayer graphene is a contender of interest for functional electronic applications because of its variable band gap due to interlayer interactions. Graphene growth on Cu is self-limiting, thus despite the fact that chemical vapor deposition (CVD) has made substantial strides in the production of monolayer and single-crystal graphene on Cu substrates, the direct synthesizing of high-quality, large-area bilayer graphene remains an enormous challenge. In order to tackle this issue, we present a simple technique using typical CVD graphene growth followed by a repetitive wrinkling-etching-regrowth procedure. The key element of our approach is the rapid cooling process that causes high-density wrinkles to form in the monolayer area rather than the bilayer area. Next, wrinkled sites are selectively etched with hydrogen, exposing a significant portion of the active Cu surface, and leaving the remaining bilayer areas, which enhance the nucleation and growth of the second graphene layer. A fully covered graphene with 78 ± 2.8% bilayer coverage and a bilayer transmittance of 95.6% at room temperature can be achieved by modifying the process settings. Bilayer graphene samples are examined using optical microscopy (OM), scanning electron microscopy (SEM), Raman spectroscopy, and an atomic force microscope (AFM) during this process. The outcomes of our research are beneficial in clarifying the growth processes and future commercial applications of bilayer graphene.

## 1. Introduction

Monolayer graphene is a desirable material for future electrical and photonic devices due to its extremely high carrier mobility and distinct one-atom-thick layered structure [1]. However, the lack of a band gap in monolayer graphene limits the exploration of graphene-based semiconductor assemblies [2]. Bilayer graphene’s extensive electrical variations have sparked a lot of attention in the realms of nano-electronics and superconductivity [3,4,5]. The most promising synthetic method for growing high-quality, large-area graphene recently has been identified as CVD, particularly on commercial Cu substrates [6,7,8]. However, a feasible synthesis technique must be created to overcome the CVD process’ self-limiting growth characteristic in order to produce bilayer graphene on a wide scale [9,10].

Density functional theory (DFT)-based first principles’ computations are currently a prognostic tool for the investigation of the growth kinetics and structure of two-dimensional materials [11,12]. Numerous theoretical models (such as the terrace growth model and the sinking growth model) have been put forth based on DFT calculations to explain the formation of bilayer graphene on the Cu substrate [13,14]. These theoretical hypotheses provide guidelines for designing experiments. Three basic avenues may be identified based on the experimental approaches used so far to fabricate large-area bilayer graphene using CVD techniques. The first involves using the van der Waals epitaxial process to develop a second layer of graphene on top of an already-existing layer [15,16]. Due to the absence of an active Cu catalytic surface, the second layer’s growth rate is regularly quite slow, resulting in bilayer areas with a diameter of a few microns. The second is to expand the size of the second layer beneath the first layer in accordance with the process of precipitation [17,18,19]. Bilayer graphene with lateral diameters up to 3 in. × 3 in. has been produced by regulating the critical equilibrium between the self-terminating process and C precipitation, for instance, where percentage-engineered CuNi alloy was most frequently used [17]. In general, the layer number generated by this technique is typically sensitive to growth conditions and because of CuNi alloy’s high corrosion resistance, the integrity is usually reduced during the graphene transfer process. Slowing down the growth of the top layer and growing two layers simultaneously can be another pathway to increase the size of bilayer graphene. This can be accomplished by intricately pretreating Cu substrates and carefully regulating the proportional pressure or concentration of the carbon source [20,21,22]. It has been stated that bilayer graphene films with up to 77% coverage on Cu can be produced using an H_2_O-assisted CVD technique. In order to facilitate the nucleation and growth of the bilayer beneath, the H_2_O strategically etches the edge of the top graphene layer, slowing the growth of the top layer [23].

Our earlier research into the wrinkling of graphene domains under rapid cooling revealed that monolayer areas wrinkled more readily than multilayer areas (≥two layers), and the boundary of the multilayer area tended to be a high-density wrinkle zone that could be selectively etched by hydrogen [24]. Based on this knowledge, we provide a novel approach to produce high-quality large-area bilayer graphene via CVD growth followed by repeated wrinkling-etching-regrowth (G-rW-rE-rG). Bilayer coverage of 78 ± 2.8% has been achieved after several growth cycles. The corresponding growth mechanism and stacking order have been studied based on the isotope-labeling technique, and the results indicate that the regrowth process of bilayer graphene follows an edge attachment and ‘nano-gap’ diffusion mechanism. Hydrogen selectively etches the wrinkled sites on the monolayer area, while preserving the bilayer area and releasing the uncovered active Cu surface. This facilitates the formation of the second graphene layer beneath. 

## 2. Experiments

### 2.1. G-rW-rE-rG CVD Growth of Large-Area Bilayer Graphene

**Figure 1:**
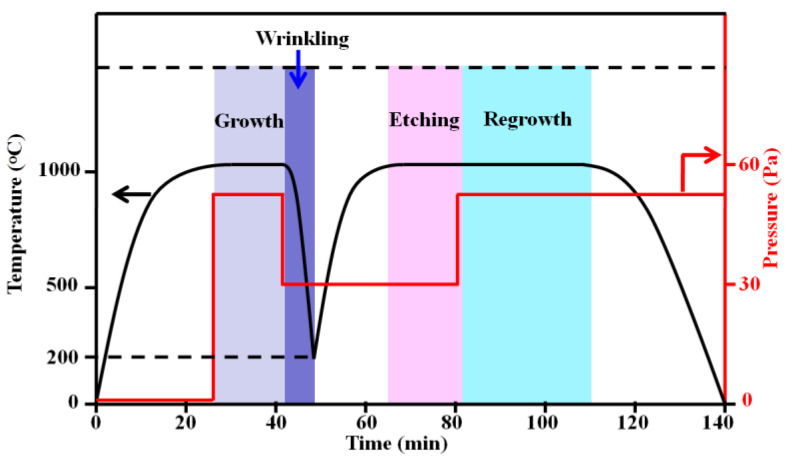
G-rW-rE-rG parameters for bilayer graphene synthesis.

The 25 μm thick commercial Cu foils (Alfa Aesar stock No. 46365) were shaped into a pocket and then pushed into a quartz tube from Xiamen G-CVD. The system was vacuumed to 0.3 Pa and then heated to the target temperature (1030 °C) in 22 min. Graphene growth was then initiated by introducing a mixture gas of 20 sccm H_2_ and 20 sccm CH_4_ for 18 min. Following CVD growth, the methane flow was halted, and the system was fast-cooled down to 200 °C under a cooling rate of ~150/min to induce wrinkles’ formation in the monolayer area. Then, we reheated the system to 1030 °C in 15 min and annealed the wrinkled graphene samples in 20 sccm H_2_ gas (~29 Pa) for 18 min to induce selective etching on the wrinkled sites. After etching, methane (4–20 sccm) was reintroduced for bilayer graphene regrowth (20 min–1 h). To obtain high-coverage bilayer graphene, four cycles of rW-rE-rG were used. Figure 1 provides a description of the experimental setup in detail. The Cu pocket was promptly removed from the high-temperature region when the reaction was finished. The PMMA-assisted (poly(methyl methacrylate)) technique was used to transfer the graphene generated on the inside surface of the Cu pocket onto target substrates such as quartz and 300 nm SiO_2_/Si substrates for further characterizations [25].

### 2.2. Characterizations

The bilayer graphene samples were examined by OM, Raman, AFM, SEM, and UV-vis spectroscopy. Studies on optical microscopy were conducted using the reflectance mode of a digital optical microscope (Metallurgical Microscope, MV5000, Nanjing Jiangnan Novel Optics Co., Ltd., Nanjing, China). SEM (Sigma, Zeiss Inc., Oberkochen, Germany) and AFM (Dimension Icon, Bruker, Karlsruhe, Germany) were used to analyze the surface morphology of the wrinkled graphene samples. By performing a Raman analysis with a Witec laser Raman spectrometer (Alpha-300, WITec Wissenschaftliche Instrumente und Technologie GmbH, D-89081 Ulm, Germany, laser excitation energy of 488 nm and resolution of ~207 nm), the structure and bonding characteristics were identified. The UV-vis spectra of graphene on quartz were collected on an Evolution220 spectrometer (Thermo Fisher Scientific, Waltham, MA, USA). 

## 3. Results and Discussion

**Figure 2:**
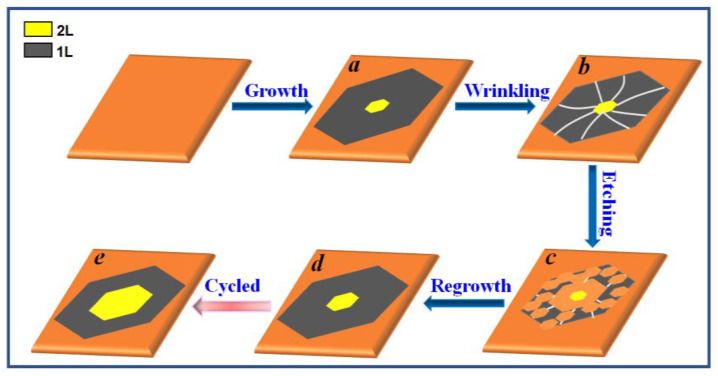
Diagram showing the G-rW-rE-rG procedure. (**a**) CVD graphene growth on Cu substrates. (**b**) Wrinkling after growth. (**c**) Hydrogen-induced etching. (**d**) Regrowth of the bilayer graphene. (**e**) Large-area bilayer graphene obtained by a few G-rW-rE-rG cycles.

Figure 2 shows the unique G-rW-rE-rG schematic diagram. First, using typical CVD growth conditions, we produced bilayer graphene on the internal surface of a Cu pocket substrate (Figure 2a). Bilayers can only make up a limited portion of the domain’s center, as previously described, because graphene development on Cu is mostly controlled by a self-terminating mechanism [21]. Then, the sample was cooled rapidly to induce wrinkle formation [26,27]. Because the thermal expansion coefficients of graphene and Cu have the opposite polarity, a residual strain will be produced by thermal quenching during fast cooling. In the presence of wrinkle formation at weakly interacting locations, CVD graphene tends to buckle by releasing strain effects. Wrinkles typically develop in the monolayer area and break on the edge of the bilayer area at a relatively low strain condition (Figure 2b) [24]. This particular layer-dependent wrinkle distribution difference determines that monolayer areas can be easily etched away due to the preferential absorbing of hydrogen at the wrinkle locations compared to bilayer areas. In order to induce selective graphene etching on monolayer portions, we switched from a growth state to an etching condition. The separation of the monolayer and bilayer areas resulted from the etching on wrinkled sites being accelerated with increasing time. Most graphene monolayer portions were eventually etched away, leaving only a few small bilayer areas behind (Figure 2c), which corresponded to the original bilayer graphene generated. After etching, we reinstated growth in the etched bilayer regions (Figure 2d). We performed a second rW-rE-rG cycle as soon as graphene had nearly covered the Cu surface. Large-area bilayer graphene was produced after multiple cycles (Figure 2e). 

**Figure 3:**
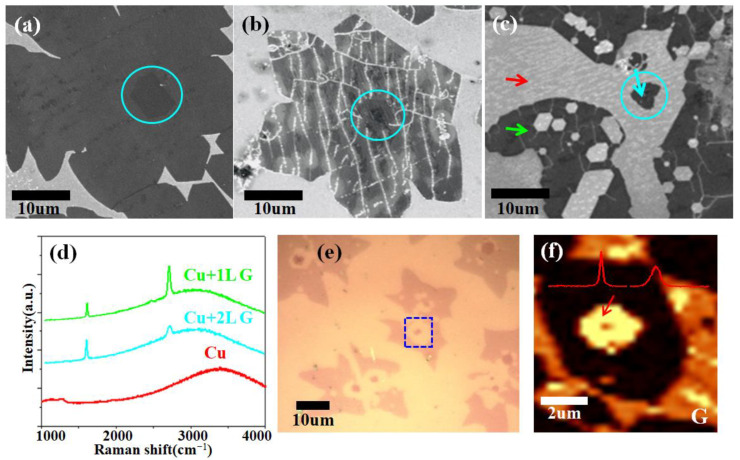
(**a**–**c**) SEM images of graphene domain on copper corresponding to initial growth (**a**), wrinkled (**b**) and etched (**c**), respectively. Regions marked by cyan circles indicate bilayers or multilayers. (**d**) Raman spectra collected from the locations marked by colored arrows in (**c**). (**e**) OM image of etched graphene samples transferred to SiO_2_/Si substrates. (**f**) Raman mapping of the G band intensity (1510−1650 cm^−1^) from the blue dot-marked area in (**e**). Inset: Raman spectrum collected from the red arrow marked area.

Figure 3a–c display the SEM analysis results that correlate to Figure 2a–c, respectively. The brightness contrast can be used to identify the layer difference, and the darker area in the center (shown by the cyan circle in Figure 3a–c) corresponds to the bilayer or multilayer [21]. Only the surface of the monolayer region and the boundary of the bilayer graphene area exhibited wrinkles with a width range of 10 nm to 60 nm (corresponding to white lines in Figure 3b; an AFM characterization is presented in Figure S1). The darker section contained no white lines. This is in line with our earlier research [24]. The H_2_-induced selective etching results are illustrated in Figure 3c. The wrinkles showed a preference for atomic hydrogen, and hexagonal etch holes frequently formed along the wrinkles. The bilayer and monolayer regions were separated as a result of etching because the boundary of bilayer graphene also functions as a high-density wrinkle zone. After etching, Raman characterization was conducted to determine the structure of the residual area. The green curve in Figure 3d confirms the presence of monolayer graphene (green arrow marked in Figure 3c, I_2D_/I_G_~1.5, full width at half-maximum (FWHM) of the 2D peak~33.5 cm^−1^) [21,25]. As highlighted by the cyan arrow in Figure 3c, the cyan curve reflects the Raman spectra of the remaining bilayer region in the center. The intensity of the G peak exceeded that of the 2D peak (I_2D_/I_G_~0.5) and the FWHM was only 53 cm^−1^, indicating the existence of AB-stacked bilayer graphene. We analyzed Raman spectra obtained from different post-etching central area locations to assess the stacking structure and number of layers. It was shown that most of the remaining central area was bilayer, with occasional multilayer structures. However, the stacking modes of bilayer structures included both AB and non-AB types (Figure S2). This probably can be attributed to the rough copper surface used (as we received copper without any pretreating) and rapid growth rate [25]. The red curve shows the Raman spectrum of the uncovered Cu, as labeled by the red arrow in Figure 3c. To further confirm the structure of the remaining area, we transferred the samples to the surface of SiO_2_/Si substrates. The thickness, as well as the homogeneity of the transferred sample, can be assessed by the color contrast under optical microscopy, with each central region of the graphene domain presenting a darker color (Figure 3e). Figure 3f shows the Raman maps (corresponding to the blue dot-marked square area in Figure 3e) of the G band (1510–1650 cm^−1^) with a varied brightness contrast, demonstrating the presence of the monolayer and bilayer areas. The remaining bilayer area in the center had a significant interlayer coupling, as evidenced by the 1.5 I_G_/I_2D_ and 56 cm^−1^ FWHM of the 2D band in the Raman spectrum (inset of Figure 3f) collected from the arrow-marked area. 

**Figure 4:**
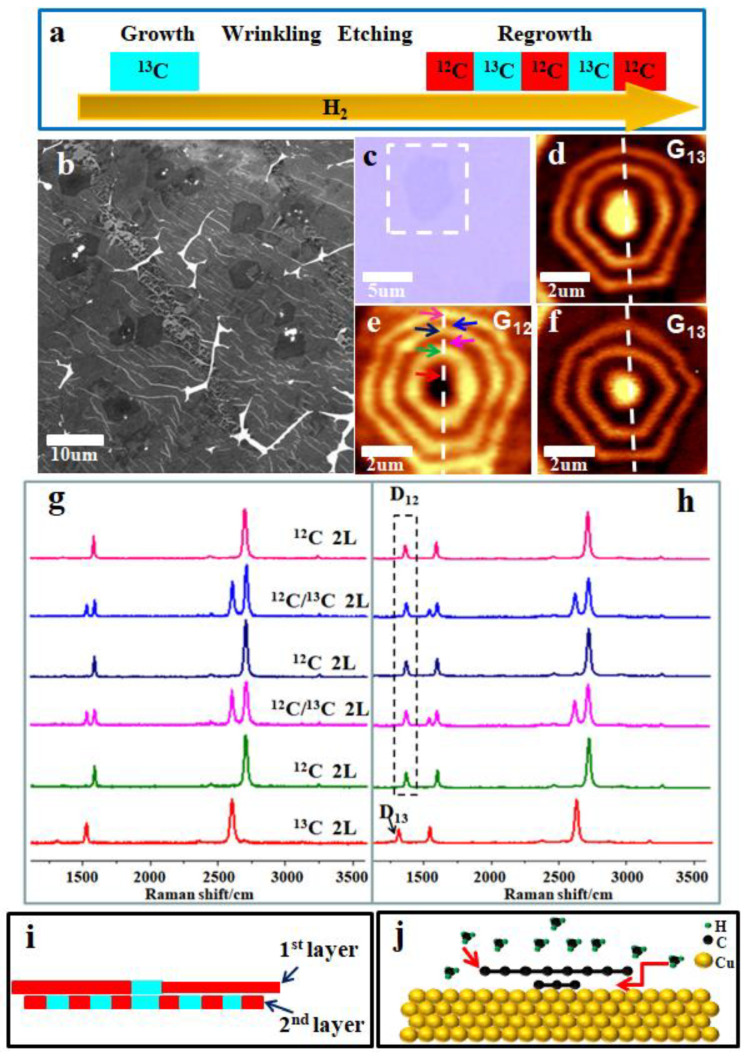
(**a**) Schematic of the pulsed carbon isotope labeling process. (**b**) SEM image of the obtained graphene samples. (**c**) OM image after transferred to the SiO_2_/Si substrates. (**d**,**e**) Raman maps of G_13_ band intensity (1480–1550 cm^−1^) and G_12_ band intensity (1550–1620 cm^−1^) from the white dotted square in (**c**). (**f**) Raman maps of G_13_ band intensity (1480–1550 cm^−1^) after O_2_ plasma etching. (**g**) Raman spectra collected from the locations along the white dotted line marked in (**e**) by arrows. (**h**) Raman spectra collected from the spots along the white dotted line marked in (**e**) by arrows after O_2_ plasma exposure. (**i**) Cross-sectional view of the graphene layer structure at the location indicated by a white dotted line in (**d**–**f**). (**j**) Schematic of growth mechanism of the bilayer graphene.

The nucleation and growth kinetics of graphene on Cu have been widely investigated and it is believed that the stable graphene nuclei’s formation only occurs with the supersaturation of active carbon atoms at the Cu surface [28,29]. This is why pre-nucleation can be used to successfully regulate the growth of two-dimensional graphene materials [30,31]. In our previous study, we performed a detailed study of graphene oxide flakes’ pre-seeded monolayer and multilayer graphene growth process by carbon isotope labeling and Raman mapping [32]. Here, to explore whether the remaining bilayer area after etching acted as pre-nucleation sites in the next regrowth step, methane and isotopically tagged methane were introduced sequentially: 5 sccm ^13^CH_4_ for 20 min in the first growth step, then 5 sccm ^12^CH_4_ for 5 min/5 sccm ^13^CH_4_ for 5 min/5 sccm ^12^CH_4_ for 5 min/5 sccm ^13^CH_4_ for 5 min/5 sccm ^12^CH_4_ for 5 min in the regrowth step, as Figure 4a illustrates (see the Methods section in SI for details). After the regrowth step was completed, the system was quickly cooled down, and the corresponding SEM image of as-grown samples is shown in Figure 4b. The wrinkles were distributed on the monolayer surface bypassing the bilayer area (darker area in the center). Then, the samples were transferred onto SiO_2_/Si wafers and the layer difference can be distinguished by the color contrast in the optical image (Figure 4c). Micro-Raman mapping was used to identify the distinctive phonon mode signatures of the isotope-engineered bilayer areas, as shown by the white dotted square in Figure 4c. Figure 4d,e show the Raman maps of the G_13_ band (1480–1550 cm^−1^) and G_12_ band (1550–1620 cm^−1^) acquired from this growth run, respectively. The successive dosing of non-labeled and subsequently ^13^C-labeled methane resulted in isotopically different graphene regions in the film because of the way graphene grows on Cu [33,34]. The center of the G_12_ map was dark, while the center of the G_13_ map was bright. Then, as ^12^CH_4_ flowed in the regrowth step instead, the bright circle appeared around the dark area in the center in Figure 2d, which proves that the residual bilayer area after etching does indeed act as a pre-nucleation site for the lateral bilayer graphene growth. Raman spectra collected from the various regions delineated by the matching colored arrows along the white dotted line in Figure 4e are displayed in Figure 4g. The red arrow in Figure 4e points to the portion of the surviving bilayer ^13^C graphene that has undergone etching. The Raman spectrum of this area (corresponded to the red curve in Figure 4g) exhibits peaks G_13_ (1520 cm^−1^) and 2D_13_ (2550 cm^−1^) with non-AB stacking features. The green curve shows the Raman spectrum of the regrowth bilayer graphene region as the green arrow marked in Figure 4e. It shows sharp G_12_ (1580 cm^−1^) and 2D_12_ (2680 cm^−1^) peaks with a G/2D ratio that is lower than 0.5, and an FWHM of 2D band of ~35 cm^−1^, indicating that the bilayer graphene generated laterally from the remaining bilayer area was also of a non-AB-stacked nature [34]. The navy curve and pink curve in Figure 4e both demonstrate the non-AB-stacked bilayer ^12^C graphene characteristic. Raman spectra from the bilayer regions in which each layer was made entirely of ^12^C or ^13^C are shown as the purple curve and blue curve, respectively. Both spectra separately display the typical bands for G_13_, G_12_, 2D_13_, and 2D_12_, and the ^13^C monolayer graphene’s Raman spectrum shows a downward shift of all Raman features in comparison to the ^12^C monolayer graphene. 

We strategically produced defects on the top-layer graphene by exposing the transferred graphene samples to O_2_ plasma for 5 s to differentiate it from the layers underneath it in an effort to further investigate the structural composition of the lateral growth bilayer graphene. After oxygen exposure, we performed Raman spectroscopy at the same locations. The Raman G_13_ map (Figure 4f) still exhibited signatures of isotope-engineered structures. As compared to the Raman spectra acquired prior to O_2_ exposure, all Raman spectra exhibited an extra resonant peak linked to the defect bands of D_12_ (~1350 cm^−1^) or D_13_ (~1300 cm^−1^) (as shown in Figure 4h), which confirms the breakage of the bilayer graphene structures. For the ^13^C graphene bilayer regions and ^12^C graphene bilayer regions, the Raman spectra showed the D_13_ band (red curve in Figure 4h) and D_12_ band (green curve, navy curve, and pink curve in Figure 4h), respectively. The purple curve and blue curve in Figure 4h display an extra resonant peak that is exclusively linked to the ^12^C defect band. This reveals that the ^12^C graphene layer was the outermost one and that only this layer was affected by the short exposure of O_2_ plasma. A D_13_ defect band would have been discovered instead if the ^13^C layer was on top of the ^12^C layer instead of the other way around. As the stacking order of the samples was not altered throughout the transfer operation, this result signifies that the adlayer was generated beneath the first graphene layer. A schematic of the cross-section view from the white dotted line marked in Figure 4d–f is presented in Figure 4i. Hence, the regrowth mechanism of lateral bilayer graphene is that the active catalytic decomposition of CHx preferentially concentrates and attaches to the edges of the remaining bilayer graphene first, the two layers grow simultaneously, and over time the top layer overgrows, resulting in a nano-gap between the top layer and the Cu substrate. After that, the active carbon atoms need to conquer an energy barrier to diffuse into the nano-gap, which lead to a decreased growth rate of the adlayer graphene, as Figure 4j illustrates. 

**Figure 5:**
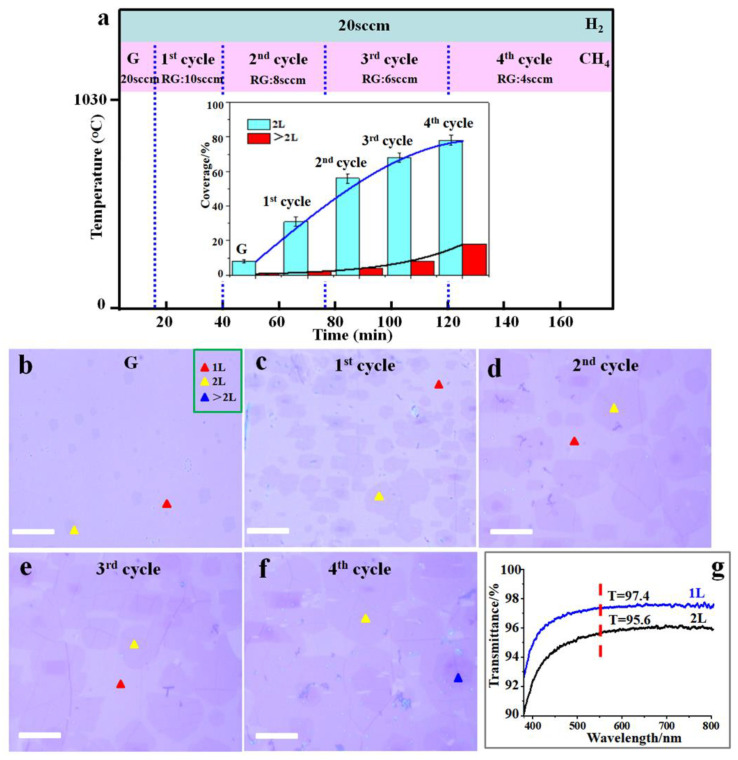
(**a**) Schematic of the G-rW-rE-rG process used for fabricating large-area bilayer graphene, with the flow rates of CH_4_ and H_2_ used. The inset shows changes in coverage of bilayer and multilayer areas as a function of growth cycles. (**b**–**f**) Optical images of the as-grown bilayer graphene transferred to SiO_2_/Si substrates in different cycles: initial growth (**b**), 1st cycle (**c**), 2nd cycle (**d**), 3rd cycle (**e**), and 4th cycle (**f**). (**g**) Transmittance spectra taken from monolayer (blue) and bilayer (black) graphene films transferred to SiO_2_ substrates. All scale bars represent 10 μm.

As demonstrated in previous studies, graphene nucleation requires a long incubation time at low methane flow rates [28,35]. We observed a significant increase in the coverage of bilayer graphene regions with decreasing CH_4_ flows (Figure S3). Therefore, to guarantee that the chemically decomposed carbon atoms attach to the edges of the remaining bilayer graphene to enlarge the area of bilayer graphene rather than aggregating to form a new nucleus, we introduced a lower rate of methane flow than in the earlier growth cycle to promote regrowth. It is worth noting that in this regrowth phase, extremely low methane flow rates are avoided. This allows for a relatively high average growth rate throughout the whole cycle (Table S2). Based on this knowledge, we modified the G-rW-rE-rG process to reduce the methane flow rate as the growth cycles increased, as illustrated in Figure 5a. Bilayer graphene was observed to be homogeneously distributed across the entire Cu foil surface (Figure S4). To study the adlayer’s coverage as well as growth rate, bilayer graphene samples with a specific cycle transferred to SiO_2_/Si substrates were investigated with optical images, where the layer variation can be observed clearly via the contrast and the monolayer area, bilayer area, and multilayer area were marked by a red triangle, yellow triangle, and blue triangle, respectively (Figure 5b–f). Rapid graphene nucleation and growth were made feasible in the first growth stage by a higher partial pressure of methane. A large number of isolated bilayer domains with an average size of ~3 μm and coverage of around 8% occurred after growth for 15 min (Figure 5b). The bilayer regions were becoming larger and the average size and coverage reached up to 10 μm and 31% after the first cycle (Figure 5c). As the cycles continued, these domains integrated with adjacent ones to create a continuous film in the second, third, and fourth cycles, and the average coverage of the bilayer reached ~56%, 68%, and 78%. The bilayer coverage increased almost linearly along with the increase in cycles (the inset of Figure 5a). When we further increased the cycle with a reduced methane flow at 4 sccm, multilayer domains started growing (Figure 5f, a multilayer coverage of 18%), which was probably caused by the precipitation of the saturated C atoms dissolved into the copper after a few growth cycles. We have also assessed the average layer number of graphene using optical transmittance. Figure 5g demonstrates that the bilayer graphene film produced using the G-rW-rE-rG approach has a transmittance of 95.6% at 550 nm, which is consistent with the value for bilayer graphene that has been published [22,23]. 

**Figure 6:**
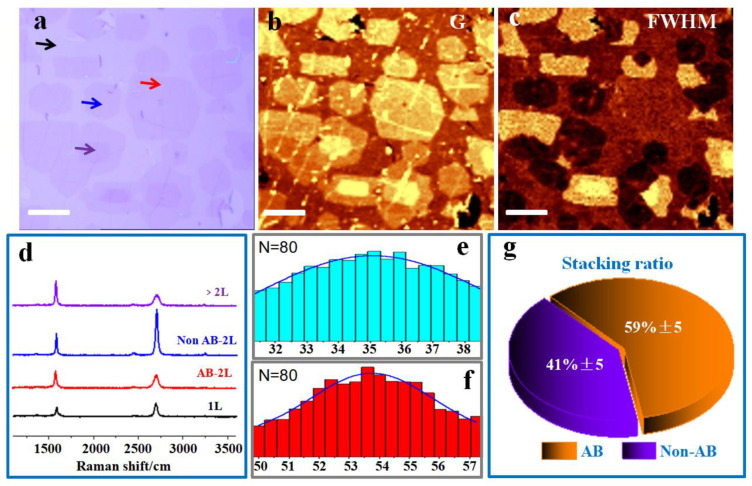
(**a**) Optical image of bilayer graphene films on SiO_2_/Si substrates. (**b**,**c**) Raman G-band map (1560–1660 cm^−1^) and FWHM of 2D band map (2650−2760 cm^−1^). (**d**) Raman spectra taken from the colored arrows marked areas in (**a**). (**e**,**f**) Histograms of the 2D FWHM of non-AB-stacked and AB-stacked bilayer graphene. (**g**) Statistics regarding the stacking ratio of the graphene sample. All scale bars represent 10 μm.

We conducted spatially resolved Raman mapping on these samples to further determine the uniformity of the obtained graphene sheets. An optical image of a portion of graphene sample grown by the third cycle transferred to SiO_2_/Si substrates is shown in Figure 6a. The color contrast of the image demonstrates that after three cycles homogenous bilayer graphene domains were achieved. Figure 6b,c display the corresponding Raman maps of the G_12_ band (1560–1660 cm^−1^) and FWHM of the 2D_12_ band (2650–2760 cm^−1^), which consist of monolayer, bilayer, and multilayer graphene. In general, the average G-band intensity is higher in the AB-stacked bilayer area than in the monolayer area. The heterogeneity of brightness in the FWHM map revealed that the bilayer graphene formed here was inhomogeneously stacked. Figure 6d presents the Raman spectra of the monolayer, AB-stacked bilayer, non-AB-stacked bilayer, and multilayer graphene. By examining the 2D/G intensity ratio and 2D-band line width, it is simpler to identify the stacking order of bilayer graphene. Our AB-stacked bilayer’s 2D/G intensity ratio was 0.5, while it was ~1.5 for non-AB-stacked, and this is comparable to the value reported in CVD epitaxial bilayer graphene [21]. The Raman peak was fitted to a single Lorentzian function to calculate the FWHM of the 2D band, and we analyzed 80 different spectra collected from the samples to evaluate the FWHM values for the AB-stacked bilayer and non-AB-stacked bilayer individually. From the resulting FWHM histogram, the average values of as-grown non-AB-stacked and AB-stacked bilayers were determined to be 35 cm^−1^ and 53.5 cm^−1^ (Figure 6e,f). Hence, combined with the analysis of the 2D/G intensity ratios, FWHM of the 2D band, and also the line shape of 2D peaks, we concluded that 59 ± 5% of the bilayer graphene film we synthesized consisted of AB-stacked layers and a fraction of bilayer graphene did not have AB stacking (Figure 6g). We believe that this is because a portion of the initially obtained bilayer graphene which served as pre-nucleation in the regrowth process was not AB stacked. (Figure S2) [32].

## 4. Conclusions

We devised a G-rW-rE-rG method to produce large-area bilayer graphene films, which exhibit bilayer coverage of 78 ± 2.8% and a transmittance of 95.6% at room temperature. The formation of large-area bilayer graphene can be attributed to edge attachment and diffusion-based growth mechanisms. Following wrinkling and selective hydrogen etching, the coverage of the top monolayer graphene decreases while a portion of the bilayer area remains, and this facilitates the nucleation and growth of the bilayers during the regrowth process. By combining the G-rW-rE-rG strategy with other methods to induce the formation of bilayer graphene with a specific stacking order in the initial growth step, it is anticipated that substantially larger or even wafer-sized homogeneous-stacked bilayer graphene may be produced. We are certain that our work will help in understanding the growth mechanisms of CVD-derived bilayer and multilayer graphene on Cu, as well as clear the way for the mass production of bilayer graphene for industrial applications.

## Data Availability

Not applicable.

## Supplementary Materials

The following supporting information can be downloaded at: https://www.mdpi.com/article/10.3390/nano13142059/s1, Methods: Growth of ^12^C/^13^C graphene. Table S1: Growth parameters for large-area bilayer graphene. Table S2: Comparison of the flow ratio, growth time, and coverage of bilayer graphene obtained on Cu by CVD (see Refs. [16,19,23,34,36,37,38,39,40,41]). Figure S1: (**a**) AFM image of wrinkled graphene transferred onto SiO_2_/Si; (**b**) The height of the thick black line marked area. Figure S2: (**a**) SEM image of H_2_-induced etching of wrinkled graphene. (**b**) Raman spectra of the different remaining graphene areas (pointed by colored arrow in (**a**)) on Cu foil. Figure S3: The influence of the methane flow rate on the growth of bilayer graphene. SEM images of bilayer graphene grown in a gas flow of 20 sccm hydrogen and (**a**) 20 sccm methane, (**b**) 15 sccm methane, (**c**) 12 sccm methane, and (**d**) 10 sccm methane, respectively. All scale bars represent 10 μm. Figure S4: (**a**) SEM image of bilayer graphene in a region of ~0.5 × 0.5 mm^2^. (**b**) Magnified SEM image of the bilayer area indicated by red rectangle in (**a**).
