# Supplementary material for: A Wrinkling and Etching-Assisted Regrowth Strategy for Large-Area Bilayer Graphene Preparation on Cu
Source: Nanomaterials (Basel). 2023 Jul 12;13(14):2059. doi: 10.3390/nano13142059 (PMC10385747; doi:10.3390/nano13142059)
Supplement: Supplementary file 1 [file nanomaterials-13-02059-s001.zip › nanomaterials-2473729-supplementary.pdf]

Supporting Information

A Wrinkling and Etching-Assisted Regrowth Strategy for Large-Area Bilayer Graphene Preparation on Cu

Qiongyu Li [1,*], Tongzhi Liu [1], You Li [2], Fang Li [2], Yanshuai Zhao [1] and Shihao Huang [1]

[1] School of Electronic, Electrical Engineering and Physics, Fujian University of Technology, Fuzhou 350118, China
[2] MIIT Key Laboratory of Semiconductor Microstructure and Quantum Sensing, Department of Applied Physics, Nanjing University of Science and Technology, Nanjing 210094, China
* Correspondence: qyli@njust.edu.cn

Methods

Growth of ^{12}C/^{13}C Graphene.

The system was first vacuumed to 0.3 pa and heated to 1030℃. Then a mixture gas of 10 sccm H_2 and 5 sccm $^{13}CH_4$ is subsequently flowed into the CVD chamber to initiate the bilayer growth (20 min). After the first growth step, the methane flow was cut off and the system was fast-cooled down to 200 ℃ in 6 min. Then the system was reheated to 1030 ℃ in 15 min and annealed in the same atmosphere for 20 min. After etching, 5 sccm $^{12}CH_4$ was first introduced for 5 min, then followed by 5 sccm $^{13}CH_4$ for 5 min/5 sccm $^{12}CH_4$ for 5 min /5 sccm $^{13}CH_4$ for 5 min / 5 sccm $^{12}CH_4$ for 5min. The regrowth time was 25 min. After growth, the system was first cooled to room temperature in H_2 atmosphere.

Table S1. Growth parameters for large-area bilayer graphene.

Cycle	Temperature (°C)	Flow ratio (H_2:CH_4)	Growth time
1st	1030	20:10	24 min
2nd	1030	20:8	36min
3rd	1030	20:6	44min
4th	1030	20:4	1h

Table S2. Comparison of the flow ratio, growth time and coverage of bilayer graphene obtained on Cu by CVD.

Temperature (°C)	Flow ratio (H_2:CH_4)	Coverage	Growth time	Reference
1050	40:1	99%	3h	36
1045	120:1	30%	2h	37
1000	40:1	50%	70min	38
1000	35:1.5	61%	180min	34
1000	500:0.5	95%	3h	19
1050	200:0.5	56%	90min	39
1040	50:2	76%	60min	23
1100	100:10	100%	70min	16
1020	20:1	93%	45min	40
1020	100:20	100%	20min	41

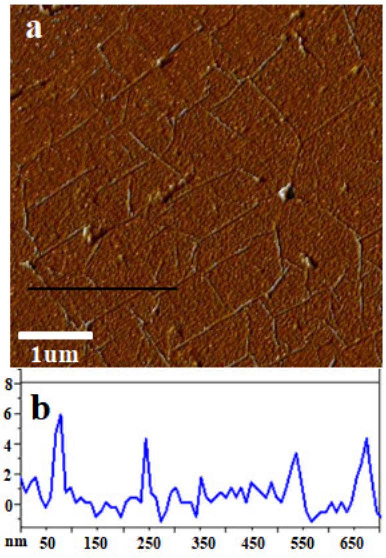

Figure S1. (a) AFM image of wrinkled graphene transferred onto SiO_2/Si; (b) The height of the thick black line marked region.

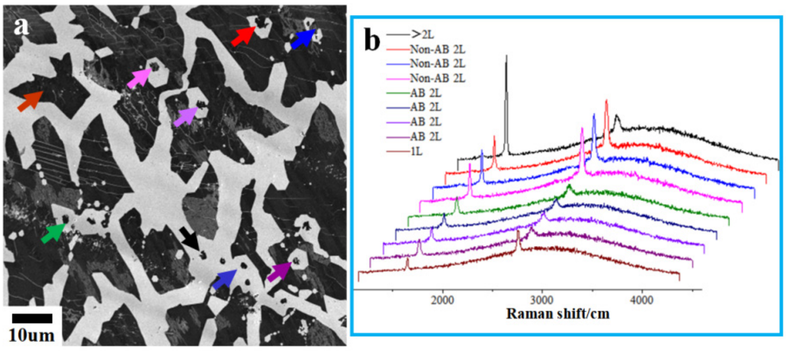

Figure S2. (a) SEM image of H_2 induced etching of wrinkled graphene. (e) Raman spectra of the different remaining graphene area (pointed by colored arrow in (a)) on Cu foil.

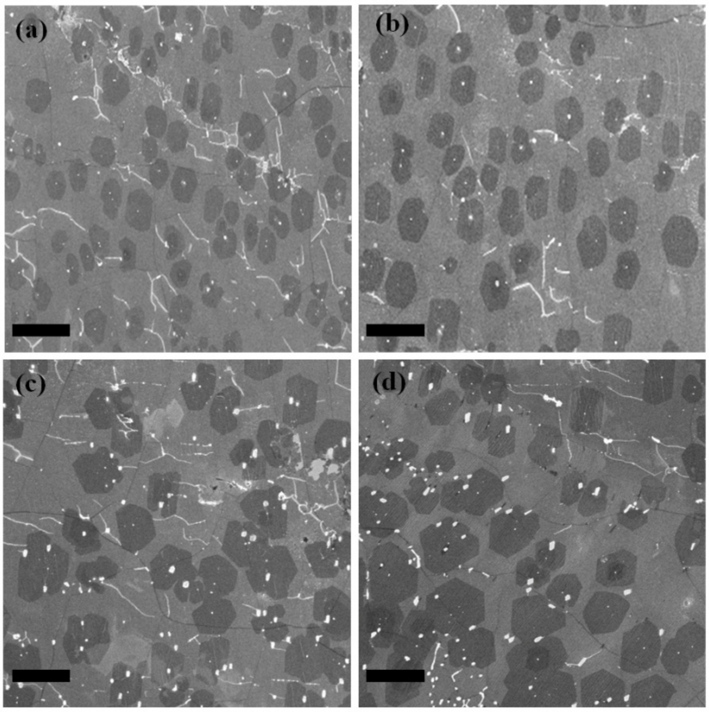

Figure S3. The influence of the methane flow rate on the growth of bilayer graphene. SEM images of bilayer graphene grown in a gas flow of 20 sccm hydrogen and (a) 20 sccm methane, (b) 15 sccm methane, (c) 12 sccm methane and (d) 10 sccm methane , respectively. All scale bars represent 10 μm.

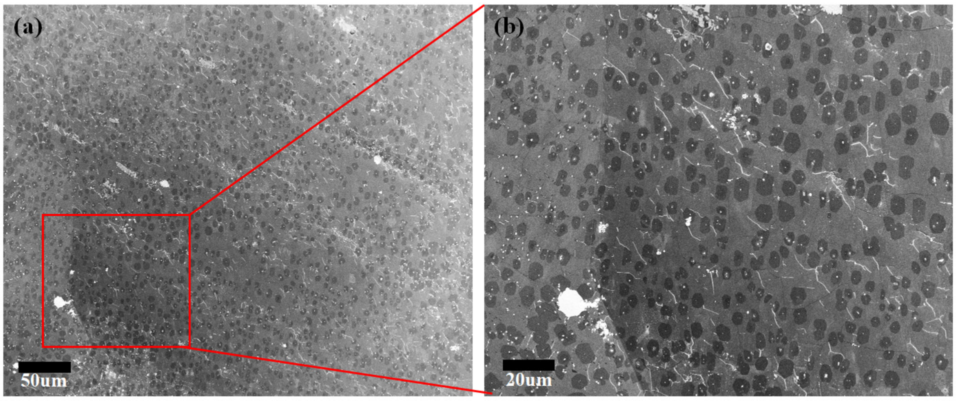

Figure S4. (a) SEM image of bilayer graphene in a region of ~0.5×0.5 mm^2. (b) Magnified SEM image of the bilayer area indicated by red rectangle in (a).
